# Biodiversity and Human Health Interlinkages in Higher Education Offerings: A First Global Overview

**DOI:** 10.3389/fpubh.2021.637901

**Published:** 2021-02-25

**Authors:** Mark Cianfagna, Isabelle Bolon, Sara Babo Martins, Elizabeth Mumford, Cristina Romanelli, Sharon L. Deem, Christina Pettan-Brewer, Daniela Figueroa, Juan Carlos Carrascal Velásquez, Cheryl Stroud, George Lueddeke, Beat Stoll, Rafael Ruiz de Castañeda

**Affiliations:** ^1^Department of Community Health and Medicine, Faculty of Medicine, Institute of Global Health, University of Geneva, Geneva, Switzerland; ^2^Global Studies Insitute, University of Geneva, Geneva, Switzerland; ^3^World Health Organization, Geneva, Switzerland; ^4^Department of Environment, Climate Change and Health, World Health Organization, Geneva, Switzerland; ^5^Institute for Conservation Medicine, St. Louis Zoo, St Louis, MO, United States; ^6^Department of Comparative Medicine, School of Medicine, University of Washington, Seattle, WA, United States; ^7^Faculty of Liberal Arts, Adolfo Ibáñez University, Santiago, Chile; ^8^One Health Colombia, Veterinary Medicine and Zootechnics Faculty, University of Córdoba, Cordoba, Colombia; ^9^One Health Commission, Apex, NC, United States; ^10^One Health for One Planet Education Initiative, Southampton, United Kingdom; ^11^Centre for the Study of Resilience, Faculty of Education, University of Pretoria, Pretoria, South Africa

**Keywords:** one health, biodiversity, education, global health, planetary health, conservation, capacity-building, climate change

## Abstract

**Introduction:** Biodiversity is inextricably linked to human health. As an important area of research of the Convention on Biological Diversity and a key avenue for the dissemination of biodiversity and health knowledge, we investigated how well-embedded biodiversity and health interlinkages are in institutional higher education offerings.

**Methods:** Using One Health education programs as a starting point, we collected a global list of institutions potentially carrying out education in the links between biodiversity and health through previously published research, academic partners of global conglomerates, and our own networks. We then analyzed the offerings from these institutions to determine the degree of integration of biodiversity and health interlinkages.

**Results:** We found 105 educational offerings in biodiversity and health interlinkages from 89 institutions in 30 countries. These were primarily found in faculties of public health, veterinary sciences, and medicine, with varying degrees of coverage of the interlinkages.

**Conclusion:** Education incorporating the links between biodiversity and health exists globally, but should be more widely integrated, particularly through inter-faculty and inter-institutional collaboration.

## Introduction

The Earth has lost 68% of its ecosystems and associated biodiversity in the last 50 years due to anthropogenic activity (1, 2). The consequences of this unprecedented global ecological and environmental disruption are now more daunting than ever before, especially if we consider the increasing number of pathogen spillover events from wildlife and domestic animals to humans (3, 4). However, outside of the One Health community, recognition of the importance of ecosystems and biodiversity for human health is often lacking. Along with climate change response, the international community needs to focus on biodiversity conservation by emphasizing holistic approaches to health such as One Health and Planetary Health (5). In 2015, the Secretariat of the Convention on Biological Diversity (CBD) and the World Health Organization (WHO), with contributions from over 100 international experts, published “*Connecting Global Priorities: Biodiversity and Human Health - A state of knowledge review”* (6). The report provides a comprehensive review of the numerous ways in which biodiversity underpins health and the health risks associated with ecosystem disruption and biodiversity loss.

The Strategic Plan for Biodiversity and its 20 Aichi Biodiversity Targets adopted by the Conference of the Parties to the CBD in 2010, provided a 10-year overarching framework for action on biodiversity to 2020 (7). Aichi Targets 1 and 19 addressed the knowledge deficiency in biodiversity and attempted to increase the awareness and appreciation in the general public as well as increase knowledge, the science base and technologies related to biodiversity. Governments have largely failed to meet the overwhelming majority of the Targets to 2020, however Targets 1 & 19 are among the few that have seen progress (8, 9). The achievements of these targets can be furthered by interdisciplinary educational institutions' integration of biodiversity and its interlinkages to human health.

In academic curricula, biodiversity is typically covered in biology and ecology courses but increasingly also in the fields of economics, geology and anthropology (10, 11). In the field of veterinary medicine, public health and more recently in areas such as tropical medicine and a few institutions of human medicine, biodiversity interlinkages are gradually being introduced as part of One Health and global health curricula, particularly in the context of zoonotic emerging infectious diseases (12, 13).

Educational programs and courses teaching One Health can now be found in faculties of veterinary medicine, public health, human medicine and applied sciences at all levels of instruction. In 2016, there were 83 academic One Health programs and courses listed in North America alone (14). Further lists of these offerings were developed in Western Europe, China, South Asia, Sub-Saharan Africa and Australia/New Zealand; but did not include South America where One Health is gaining considerable importance (14–19).

With the need for more scientific, political and societal attention to the interlinkages between biodiversity and health in order to prevent future pandemics and reduce environmental disruption (4), we set out to explore if, where, and to what extent, biodiversity and health interlinkages are covered in higher education courses, programs, modules and certificates (‘offerings', hereafter) worldwide and in high biodiversity areas (biodiversity hotspots), in particular (20).

## Methodology

As biodiversity and its links to health is part of a wide One Health approach, we first collected lists of offerings from institutions teaching One Health as identified in previous publications (14–19, 21).

Second, Partner institutions of internationally recognized platforms including One Health Commission (www.onehealthcommission.org), Consortium of Universities for Global Health (www.cugh.org), EcoHealth Alliance (www.ecohealthalliance.org) and Planetary Health Alliance (www.planetaryhealthalliance.org), were screened for offerings with biodiversity and health interlinkages.

Thirdly, additional programs and institutions were added through a web search in English using Google between May and July 2020 using the following keywords: “One Health education,” “One Health program,” “One Health course,” “One Health training,” “planetary health education,” “planetary health program,” “planetary health course,” “planetary health training,” “biodiversity and health” “biodiversity and health training” “biodiversity and health education” “biodiversity and health course,” “biodiversity and health program.” Major massive open online course (MOOC) platforms, namely Coursera, Udemy, EdX, FutureLearn and Udacity, were also searched using the following keywords: “One Health,” “planetary health,” “biodiversity and health.”

Lastly, to identify further institutions and their offerings, we performed consultations involving experts from One Health Commission, EcoHealth Alliance, and One Health Latin America and the Caribbean. Additional One Health experts in the authors' networks and the network of these were consulted. Overall, we reached out to 23 experts and spoke to 14. Through these consultations, institutions and programs in French, Spanish and Portuguese were added. For each institution listed, program descriptions, course descriptions and course syllabi (where available) were analyzed for offerings which included biodiversity and health interlinkages.

Six key biodiversity and health interlinkages are highlighted in the CBD/WHO State of Knowledge Review: ecosystem services (including pollination, food security and availability, nutrition, water quality, and air quality); climate change adaptation and disaster risk reduction (DRR); the human microbiome; traditional medicine/pharmaceuticals; spiritual, cultural and physical well-being; and emerging infectious diseases (EIDs).

Any offering which included explicit mention of one or more of these interlinkages was included in our offerings dataset. Offerings had to be available yearly with the same or similar topics, therefore one-off seminars and workshops were not included. Institutions and their offerings were excluded from in-depth review if no formal mention of training or education in biodiversity and health interlinkages or One Health was listed. Offerings from institutions which had indirect or unclear offerings were included in the mapping to emphasize regional distribution and as areas for further incorporation and capacity-building. Indirect offerings describe offerings which cover topics related to biodiversity and health interlinkages, but where there is no explicit mention of their interlinkages (ex. food safety, zoonoses, infectious disease). Unclear offerings describe offerings in which inadequate information was available to confirm coverage of interlinkages, but based on the title of the offering, it is plausible that there is coverage (ex. Master of One Health at University of Alaska Fairbanks).

## Results

A total of 341 institutions worldwide were screened for inclusion, with 219 included for in-depth review. These institutions were found through previous publications (*n* = 96), international platforms (*n* = 213), Google and MOOC platform searches (*n* = 13), and expert consultations (*n* = 19).

**Figure 1:**
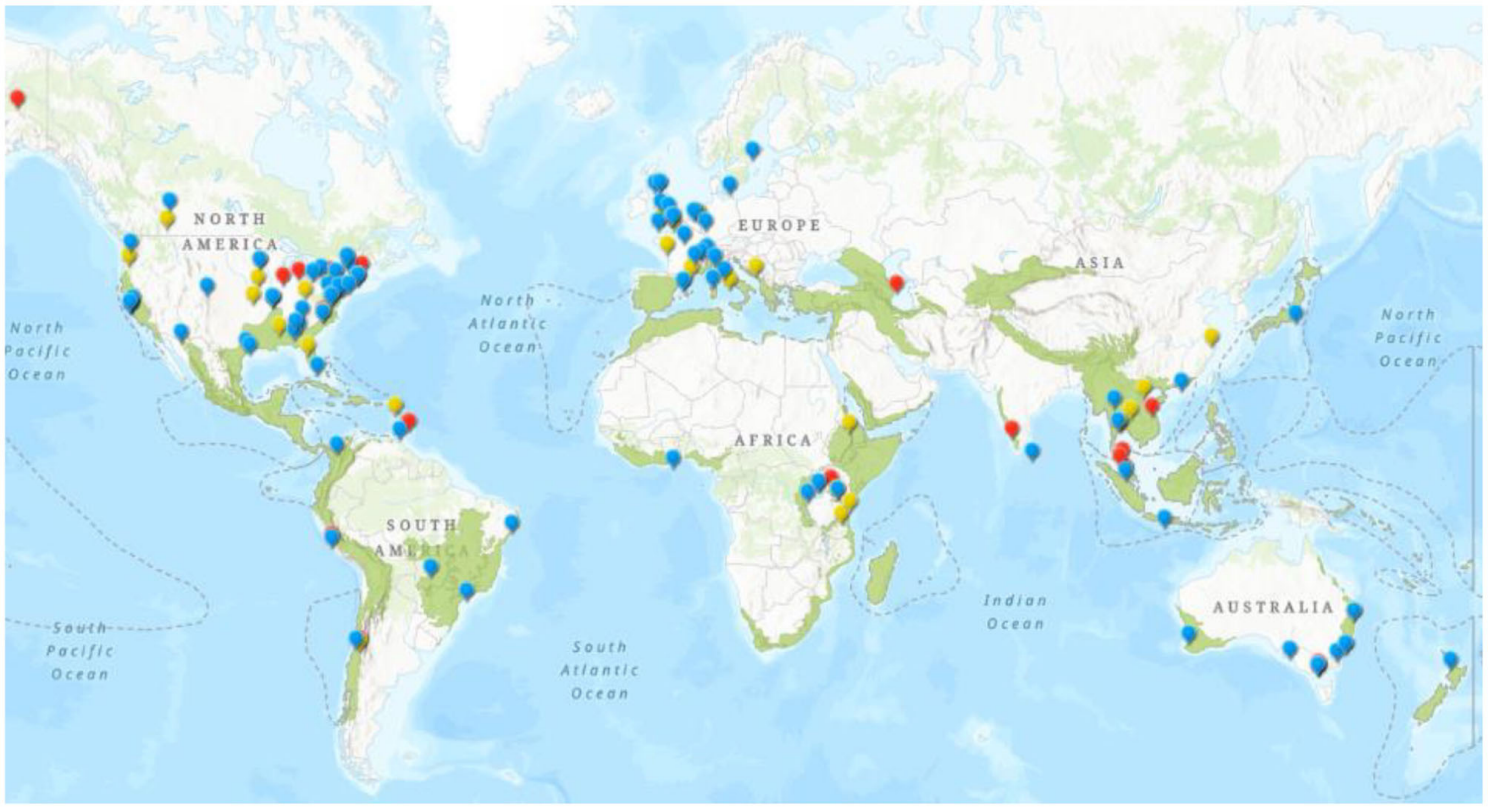
Distribution of institutions teaching biodiversity and health interlinkages (blue icons, *n* = 88). Yellow icons represent those with indirect offerings (*n* = 28); red icons, unclear offerings (*n* = 18). Distribution is overlaid on biodiversity hotspots (22).

From the 219 institutions reviewed, 105 offerings from 89 institutions and 30 countries were included in the dataset (Supplementary Table). Our data showed an important regional imbalance toward North America and Europe, where 68% of the institutions teaching biodiversity and health interlinkages are found. Oceania and Asia represented another 20%, leaving 12% of the programs split between Africa and Latin America and the Caribbean (Figure 1). Among the institutions included in our findings, 30 (34%) of them are located within biodiversity hotspots and 62 (70%) are in countries with biodiversity hotspots, as defined by Myers et al. (20) (over half of these being in the United States of America) (See Figure 1).

Offerings were found primarily in public health, veterinary medicine and human medicine faculties (22, 19, and 15%, respectively). Other faculties ranged from anthropology to forestry to sustainable development. The offerings targeted the spectrum of higher education, from open-access online courses to undergraduate, graduate, post-graduate, doctorate, and post-doctoral offerings, to continuing education for professionals. Most common were graduate-level offerings (50%), followed by undergraduate offerings (20%). There were some offerings available to professionals, typically from the public health and veterinary field. Often these were formatted as online continuing education modules or intensive short courses.

**Figure 2:**
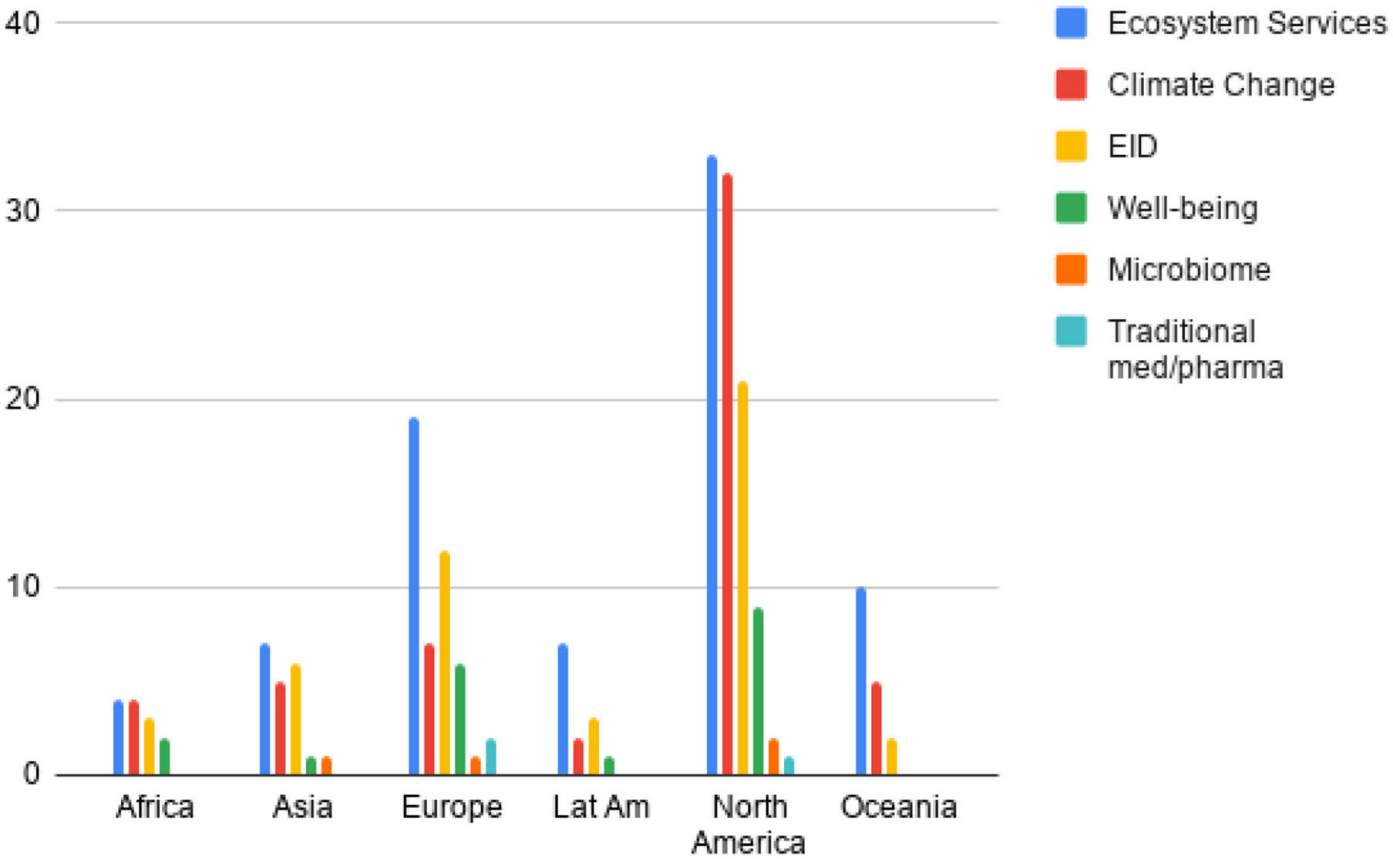
Coverage of biodiversity and health interlinkages throughout offerings by region (LatAm, Latin America and the Caribbean; EID, Emerging Infectious Diseases).

The majority of offerings covered only one or two of the biodiversity and health interlinkages (82 of 105, or 78%), while just six of them covered four of these. Encouragingly, the offerings covering three to four interlinkages (22%) came from a wide variety of faculties including human and veterinary medicine, public health, environmental science and biology. The most common interlinkages covered were ecosystem services [79 of the 105 offerings (75%)] and climate change adaptation & DRR (51%). Biodiversity and EIDs (45%) was often offered alongside ecosystem services and/or climate change. Topics such as spiritual, cultural and physical well-being, the human microbiome and traditional medicines/pharmaceuticals, all recognized as relevant areas of intersection in the State of Knowledge review, were not well-represented across the institutions (24% combined) (Figure 2).

## Discussion

There is an apparent geographical bias in institutions teaching biodiversity and health interlinkages. The majority of institutions in our dataset are based in Western Europe and North America. There is also a bias toward certain biodiversity and health interlinkages, notably ecosystem services and climate change. Many of the offerings covered only a small number of the interlinkages searched for, but offerings were found in a wide variety of faculties.

Our data show that biodiversity and health interlinkages are taught most often in public health offerings. This is likely due to the longer history of recognition of the interdisciplinary nature of public health (23). Schools of public health tend to promote inclusion of students from a variety of disciplines including physicians, nurses, veterinarians, biologists, psychologists, economists, lawyers etc. Schools of public health offering teaching in biodiversity and health interlinkages (often embedded in One Health-focused courses and programs) have some of the most comprehensive programs we found, likely because public health has long recognized the necessity for interdisciplinary and cross-sectoral collaborations and the development of a diverse workforce with knowledge of current biodiversity and health challenges (24). In our results, all six of the biodiversity and health interlinkages were found in at least one offering from a school of public health. This breadth of coverage of biodiversity and health interlinkages, and the diversity of student's backgrounds and experience in public health programs, currently makes them particularly well-suited to fostering leadership in combined biodiversity and health education.

Veterinary and human medicine programs tend to be very clinically focused and there are associated challenges of finding the space within an already well-established curriculum to incorporate new topics and content (25). Suggestions to better incorporate these concepts into veterinary education include pre-clinical education (where applicable), common coursework with medical and science students and interdisciplinary faculty working across multiple domains (26–28). These ideas do not represent fundamental changes in veterinary education, but applied learning strategies that can be introduced without overhauling existing programs. Indeed, population biology is an overarching theme in veterinary medical education, and thus biodiversity could also be included. In human medicine, physicians are rarely trained to understand, or to inform the public, about the interlinkages between biodiversity and human health, or how to apply nature-based solutions or ecosystem services in their clinical practice (29). Medical school curricula are highly standardized and clinically focused. There is often little room for advancement in more general themes and systemic approaches to health outside of the clinical setting. Clinical, rather than population level, applications of biodiversity and health interlinkages may be a way to achieve integration into medical programs (30). These applications can include the contribution of biodiversity to dietary quality and nutritional assessment, green prescriptions and conservation psychology (31–33). These are all examples of interlinkages that can be included within specialized courses already integrated in human medical curricula. However, for most biodiversity and health interlinkages, population-level applications are of highest importance. Other research suggests looking to external bodies and elective courses for integration of these interlinkages (29, 34). While simpler, this would mean only a small proportion of physicians would be trained to acknowledge and apply biodiversity and health interlinkages into their practice.

The interest of integrating topics and approaches rather than separate disciplines in higher education course offerings is that there is no need to shoehorn another course into an already packed curriculum. Having learning outcomes such as values, knowledge and skills in an approach can help with integration in health professional education (35). These topics can be included throughout educational programs as case studies, individual lectures and mandatory readings to support what is already being taught, allowing practitioners to have a more complete view of upstream drivers of health. Every medical program discusses infectious disease, so emphasis should be also placed on how ecosystem disruption and degradation can play a significant role in the emergence and re-emergence of infectious disease, particularly considering the growing number of zoonotic spillover events. This is of particular interest for medical faculties located in hotspots of disease emergence (36). Key areas for this include Latin America, West Africa and Southeast Asia, where almost every country has areas with high biodiversity and high risk of infectious disease emergence. Yet our dataset showed only a small number of offerings covering biodiversity and health interlinkages in Latin America, with only two highlighting EIDs. Future medical practitioners should be made aware of the risks associated with EIDs and the need for cross-sectoral surveillance in collaboration with veterinarians and other professionals.

Capacity-building projects to train professionals in One Health approaches can be a way to build regional and national resilience while preventing the major drivers of biodiversity loss and its effects on health (37). In our search, we found offerings not only for students but interdisciplinary high-level training for professionals which is key for region-specific, adaptable education (see Table 1). Local collaborations such as communities of practice are a way to encourage peer-to-peer education and research as well as capacity- building in education (38). Collaborations like these between faculties, universities, and development organizations allow for wider integration of biodiversity and health interlinkages and are key for distribution of knowledge (39). This is also an opportunity to address the regional institutional bias. Institutions with established teaching in the interlinkages of biodiversity and health should develop further partnerships with institutions in other areas of the world, particularly those in biodiversity hotspots, to build capacity and share knowledge (See Figure 1). There are examples of this working in the past (40) and One Health University networks in Southeast Asia and Africa, as well as One Health groups in Latin America, are great examples of current collaborations working to establish a stronger One Health workforce.

**Table 1:** Number of educational offerings per region separated according to target level of education and faculty offering the education opportunity.

	Target Level of Education^*^			**Faculty**			
	**Undergraduate**	**Graduate**	**Professional Ed**.	**Public health**	**Veterinary**	**Medicine**	**Other**
Africa	0	4	2	2	0	0	4
Asia	1	6	2	3	2	2	2
Europe	2	20	3	1	5	9	12
Lat Am	3	3	3	1	3	1	2
North America	15	29	4	16	7	7	17
Oceania	4	8	0	1	4	0	6

* *some offerings open to multiple levels of education*.

Although more prevalent in human and animal sciences, our data shows that these topics can also be found in faculties of science, agriculture, forestry, environment, development, public affairs, arts and anthropology. This variety of faculties, with many being interdisciplinary, shows that teaching in biodiversity and health interlinkages is available to a wide range of learners with many different backgrounds. It also allows for inter-faculty collaboration and mobility of students across faculties for fortified learning experiences (30, 41, 42). This demands institutional capacity and the willingness to incorporate the necessary reform of funding for interdisciplinary research and educational offerings (40). It also requires shifting away from purely curative approaches to health, toward more comprehensive approaches also focused on prevention. It is through this collaboration and adaptability that ‘sustainable curricula' can be developed (43, 44). Whether on a global scale with large institutional partnerships or a local scale with inter-faculty collaboration, education in biodiversity and health interlinkages can have a much wider reach.

There is also opportunity for broader incorporation of the interlinkages through the expansion of One Health research already existing in several institutions. Research like that by Togami et al. (45), which lays out step-by-step approaches to incorporating One Health concepts into academic programs, are very informative for institutions and can aid them in adjusting complicated curricula. As shown, multiple institutions around the world are integrating biodiversity and health interlinkages into their teachings in a variety of disciplines. However, more can be done to integrate these concepts into a wider variety of programs and to reach a wider audience. The understanding of human connection to the natural world has been brought to the forefront due to the COVID-19 pandemic. Studies have shown large increases in the use of green spaces and searches for the connection of the current crisis to nature (46–48). COVID-19 has provided us with a reinforcement of the need for more work to protect biodiversity for our own health and may create more opportunities for education as the world adapts to a new understanding of ecosystem function. Education, with particular emphasis on the unifying One Health concept, remains a key to global sustainability (44). As the world moves into another decade, one the UN has described as the UN Decade on Ecosystem Restoration (49), teaching in biodiversity and its links to global health and well-being should be expanded throughout higher education offerings.

## Limitations

This being a first preliminary overview, with a simple, yet rigorous methodology, we could not comprehensively cover all offerings worldwide. Some may have been missed in the initial searches due to language or availability of information on the web or in published literature. For instance, ecohealth was not used as a search keyword in the methodology due to initial searches turning up few results and opportunities in ecohealth education being captured through partner institutions of the EcoHealth Alliance. Our consultations were also limited to Latin America, partly due to the research focus of the special issue of this journal. Further consultation with colleagues in Europe, Africa and Asia would surely reveal other offerings. We also recognize the importance of workshops, conferences and seminars as important capacity-building activities which can lead to deeper institutional integration of the interlinkages, especially for in-service professionals. This is beginning to show in Latin America as One Health activities grow through institutions with strong One Health leadership. Next steps will include more localized surveys of faculties in order to discover more educational offerings. In fact, we have already started this in Brazil and other Latin American countries. Further discussions and a chance to distribute surveys such as Omrani et al. (50) did for climate change with medical students will allow for a more complete picture of higher education offerings in the interlinkages of biodiversity and health.

## Conclusion

This preliminary overview has shown that biodiversity and health interlinkages are increasingly being integrated into a wide variety of higher education institutions and their educational offerings worldwide. Further integration with wider coverage of biodiversity and health interlinkages is especially needed in institutions within biodiversity hotspots as these are the places most likely to experience the direct health effects of biodiversity loss. This can be accomplished through inter-faculty and inter-institutional collaboration and restructuring of funding for interdisciplinary research. With growing attention to the interlinkages between biodiversity and human health through recent pandemics and the decade on ecosystem restoration, we hope to see greater integration of these vital links into higher education.

## Data Availability Statement

The original contributions presented in the study are included in the article/Supplementary Material, further inquiries can be directed to the corresponding author/s.

## Author Contributions

MC main writing of article, collected and analyzed data, hosted weekly meetings, identified, and completed consultations. RRdC head supervisor of project, coordinating author, concept development, contributed to weekly meetings, provided connections to network, and reviewed text. BS reviewed text and integral supporter of authors in Geneva. GL connected authors with network and reviewed text. JV added data to dataset in Spanish and helped develop survey for Latin America. DF added data to dataset in Spanish and reviewed text. CS connected first author with network and helped to fill in areas of data deficiency in Latin America. CP-B added data to dataset in Portuguese, connected authors to network, and developed survey for LatAm. SD added data to dataset and reviewed and revised manuscript text providing further references and perspective. CR consult on project, reviewed and revised text, adding perspective, and further references. EM reviewed and revised multiple drafts of text, making significant changes. SM concept development, review and revision of all drafts of text, and consultation throughout process. IB regular advisor on project, concept development, added data to dataset in French, and contributed to weekly meetings. All authors contributed to the article and approved the submitted version.

## Conflict of Interest

The authors declare that the research was conducted in the absence of any commercial or financial relationships that could be construed as a potential conflict of interest.
