# Supplementary material for: Biodiversity and Human Health Interlinkages in Higher Education Offerings: A First Global Overview
Source: Front Public Health. 2021 Feb 25;9:637901. doi: 10.3389/fpubh.2021.637901 (PMC7946966; doi:10.3389/fpubh.2021.637901)
Supplement: Supplementary file 1 [file Data_Sheet_1.PDF]

Supplementary Material: Institutions with education in biodiversity and health interlinkages

*ES - Ecosystem services, CC - Climate change adaptation and disaster risk reduction, EID - emerging infectious disease, WB - physical, mental and cultural well-being, Pharma - Traditional medicine and biodiversity for pharmaceuticals, MB - microbial diversity and non-communicable diseases

Program/Course Title (language of instruction if not English)	Region	Country	Institution	Faculty	Delivery	Length	Target group	#of Concepts	Concepts Covered
Food Security Major (extension of Sun-Yat Sen Uni Biology Major)	Oceania	Australia	Griffith University	Environmental Futures Research Institute	in-person	3 years	undergraduate	1	ES
Global Change and Human Health course	Oceania	Australia	University of South Australia	Faculty of Clinical and Health Sciences	in-person or online	1 semester	undergraduate	3	ES, EID, CC
Master of Public Health (focus on the ecological model of public health)	Oceania	Australia	University of Canberra	Faculty of Health	in-person or online	1-2 years	graduate	1	ES
Planetary Health Course in MPH	Oceania	Australia	University of Technology Sydney	Graduate School of Health	in-person	1.5-3 years (program)	graduate	1	ES
Course: Global Challenges: Planetary Health; One Health	Oceania	Australia	University of Sydney	School of Public Health	in-person and online	1 semester	undergraduate; graduate	2	ES, CC
Master of Food Security	Oceania	Australia	Murdoch University	School of Veterinary and Life Sciences	in-person	2 years	graduate	1	ES
Master of Veterinary Studies (Conservation Medicine)	Oceania	Australia	Murdoch University	School of Veterinary and Life Sciences	online	1 year	graduate vet	1	CC
One Health courses in Vetmed program	Oceania	Australia	Murdoch University	School of Veterinary and Life Sciences	in-person	5 years (program)	Vet	1	CC, ES
Master of Environment and Sustainability	Oceania	Australia	Monash University	Sustainable development institute	in-person	2-4 years	graduate	3	ES, CC, EID
Professional Master of One Health (Portugese)	Latin America and the Carribean	Brazil	Universidad Federal Rural de Pernambuco	Department of Veterinary Medicine	in-person	2 years	graduate/profes sionals	1	ES
PhD in Global Health and Sustainability	Latin America and the Carribean	Brazil	University of Sao Paulo	Faculty of Public Health	in-person	n/a	post-graduate	1	ES
Bachelor of Veterinary Medicine	Latin America and the Carribean	Brazil	Federal University of Mato Grosso do Sul	Faculty of Veterinary Science and Animal Husbandry	in-person	5 years	vet	1	ES
Graduate course in planetary health	North America	Canada	University of Toronto	Dalla Lana School of Public Health	in-person	12 weeks	graduate	2	ES, CC
Bachelor of Arts Ecological Determinants of Health	North America	Canada	McGill University	Faculty of Arts	in-person	4 years	undergraduate	3	ES, EID, CC
MOOC (Massive Open Online Course) - 'Wicked Problems, Dynamic solutions: The Ecosystem Approach and Systems Thinking'	North America	Canada	Concordia University/UNEP	n/a	online	own pace	anyone	3	ES, CC, EID
One Health Course	North America	Canada	University of Alberta	School of Public Health	in-person	1 semester	undergraduate/ graduate	2	ES, EID
Honours Specialization in One Health as part of Bachelor of Medical Sciences	North America	Canada	Western University	Shulich School of Medicine and Dentistry	in-person	4 years	undergraduate	2	ES, CC
Innovative Strategies for the Challenges of Global Change	South America	Chile	Adolfo Ibáñez University	Faculty of Liberal Arts	in-person	1 semester	undergraduates	1	ES
Diploma in Wildlife Medicine (Spanish)	South America	Colombia	University of Cordoba	Veterinary Medicine and Zootechnics Faculty	in-person	1 semester	undergraduate, graduate	4	ES, CC, EID, WB
Master of Global Health	Europe	Denmark	University of Copenhagen	Faculty of Health and Medical Sciences	in-person	2 years	graduate	3	ES, CC, WB
One Health international summer course	Europe	Denmark	University of Copenhagen	Faculty of Health and Medical Sciences	blended (online and in-person requirements)	5 weeks	graduate, postgraduate, professional	1	EID

Supplementary Material: Institutions with education in biodiversity and health interlinkages

*ES - Ecosystem services, CC - Climate change adaptation and disaster risk reduction, EID - emerging infectious disease, WB - physical, mental and cultural well-being, Pharma - Traditional medicine and biodiversity for pharmaceuticals, MB - microbial diversity and non-communicable diseases

Program/Course Title (language of instruction if not English)	Region	Country	Institution	Faculty	Delivery	Length	Target group	#of Concepts	Concepts Covered
Professional diploma 'One Health in Practice'	Europe	France	école nationale des services vétérinaires	école interne de vetagro-sup	in-person	3 weeks	professionals	2	WB, ES
Planetary Health course in Master of Public Health	Europe	France	EHESP Paris	Public Health	in-person	2 years (program)	graduate	3	EID, ES, MB
PhD One Health and Urban Transformation	Europe	Germany	Univeristat Bonn	Centre for Development Research	in-person	3-6 years	post-graduate	2	ES, EID
MPhil Sustainability Science	Africa	Ghana	University of Ghana	Dpt. of Biological, Environmental and Occupational Health	in-person	2 years	graduate	1	ES
One Healh Public Health Applications; One Health, One Medicine: A Global Health Approach course	Latin America and the Carribean	Grenada	St. Georges University	SGU Online	online	own pace	anyone	2	EID, ES
Master of Public Health	Asia	Hong Kong	Chinese University of Hong Kong	School of Public Health and Primary Care	in-person	1-2 years	graduate	2	EID, ES
IndoHUN, DVM One Health/EcoHealth module	Asia	Indonesia	Gadjah Mada University	Faculty of Veterinary Medicine	in-person	1 semester (as part of vet med program)	vet	2	ES, EID
Master in Biodiversity and Environmental Health	Europe	Italy	University of Siena	Department of Life Sciences	in-person	2 years	graduate	2	ES, Pharm
Master Environmental Change and Global Sustainability	Europe	Italy	State University of Milan	Faculty of Science and Technology	in-person	2 years	graduate	2	CC, ES
Planetary Health Course in MPH-GH	Asia	Japan	Tokyo Medical and Dental University	Graduate School of Medical and Dental Sciences	in-person	2 years (program)	graduate	2	CC, ES
AFROHUN; One Health Horn; Master in Climate Change Adaptation	Africa	Kenya	University of Nairobi	Institute for Climate Change and Adaptation	in-person	2 years	graduate	1	CC
Master of Science in Public Health (Global Health)	Asia	Malaysia	University of Cyberjaya	Faculty of Medicine	in-person	1-2 years	graduate	1	CC
Masters in One Health	Europe	Netherlands	Utrecht University	Faculty of Medicine School of Life Sciences	in-person	2 years	graduate	2	EID, ES
Master of Biology in Biodiversity and Sustainability	Europe	Netherlands	Leiden University	Faculty of Science	in-person	2 years	graduate	2	ES, Pharm
Bachelor of Science in Ecology and Sustainability	Oceania	New Zealand	Massey University	College of Sciences	in-person	3 years	undergraduate	1	ES
Research partnerships, trained foreign students in Asia in One Health in previous years,<br>Master of veterinary studies (veterinary public health)	Oceania	New Zealand	Massey University	School of Veterinary Science	in-person	1.5-4 years	Vet	1	ES
Climate Change and Health Symposium	South America	Peru	Instituto Medicina Tropical Alexander von Humboldt	Medicine	in-person	2 days	professionals	3	CC, ES, EID
One Health Concentration	Africa	Rwanda	University of Global Health Equity	N/A	in-person	2 years	graduate	3	ES; CC, EID
Master of Public Health	Asia	South Korea	Seoul National University	Graduate School of Public Health	in-person	2-4 years	graduate	2	CC, MB
Undernutrition and Food Security Course	Europe	Spain	Barcelona Institute for Global Health	Global Health	in-person	2 weeks	graduate	1	ES
Master Zoonoses and One Health	Europe	Spain	Universitat Autonoma de Barcelona (UAB)	Veterinary School	in-person	1 year	graduate	1	ES

Supplementary Material: Institutions with education in biodiversity and health interlinkages									
*ES - Ecosystem services, CC - Climate change adaptation and disaster risk reduction, EID - emerging infectious disease, WB - physical, mental and cultural well-being, Pharma - Traditional medicine and biodiversity for pharmaceuticals, MB - microbial diversity and non-communicable diseases									
**Program/Course Title (language of instruction if not English)**	**Region**	**Country**	**Institution**	**Faculty**	**Delivery**	**Length**	**Target group**	**#of Concepts**	**Concepts Covered**
Climate Change and Health Intensive Short Course	Asia	Sri Lanka	University of Peradeniya	Centre for Environmental Studies	in-person	2 days	professionals	2	CC, ES
Master of Outdoor Environments for Health and Well-being	Europe	Sweden	Swedish University of Agricultural Sciences	Agriculture	distance learning	2 years	graduate	1	WB
HV0178 Humans, Animals, Ecosystems – the One Health Approach in a Sustainable Global Animal Production	Europe	Sweden	Swedish University of Agricultural Sciences	Agriculture	In-person	one semester	undergraduate	1	EID
MOOC - Global Health at the Human, Animal, Ecosystem Interface	Europe	Switzerland	Université de Genève	Faculty of Medicine	online	own pace/8 weeks	anyone	4	EID, ES, WB, CC
One Health course in Master of Global Health;	Europe	Switzerland	Université de Genève	Faculty of Medicine	in-person	2 years (program)	graduate	4	EID, ES, WB, CC
MPH Global Health EcoHealth Concentration	Asia	Thailand	Thammasat University School of global studies	Faculty of Public Health	in-person	1-2 years	graduate	2	ES, EID
PhD Global Health One Health concentration	Asia	Thailand	Thammasat University School of global studies	Faculty of Public Health	in-person	4 years	post-graduate	2	ES, EID
THOHUN, Master of Bioveterinary sciences	Asia	Thailand	Master Environmental Change and Global Sustainability	Faculty of Veterinary Medicine	in-person	1-2 years	graduate	1	EID
One Health Modules	Asia	Thailand	SEAOHUN	N/A	online	own pace	professionals	4	EID, WB, ES, CC
Through Africa One Health University Network (formerly One Health Central and Eastern Africa (OHCEA)) runs online One Health modules	Africa	Uganda	Makerere University	School of Public Health	online	own pace	professionals	4	ES, EID, WB, CC
MSc Environment and Human Health	Europe	UK	University of Exeter	College of Medicine and Health	in-person	1-3 years	graduate, science	1	WB
MSc Biodiversity, Wildlife and Ecosystem Health	Europe	UK	University of Edinburgh	Edinburgh Medical School, Biomedical Sciences	online	3 years part-time	graduate	1	ES
PhD in One Health	Europe	UK	University of Liverpool	Faculty of Health and Life Sciences	in-person	2-6 years	post-graduate	2	ES, EID
MSc Biodiversity, Conservation and Epidemiology	Europe	UK	University of Glasgow	Institute of Biodiversity, Animal Health and Comparative Medicine	in-person	1 year	graduate	2	ES, EID
BASc Global Challenges; Planetary Health	Europe	UK	Brunel University London	Institute of Environment, Health and Societies	in-person	3-4 years	undergraduate	1	ES
MSc Environmental Leadership and Management	Europe	UK	University of Nottingham	Institute of Leadership and Management	in-person	1 year	graduate	2	CC, ES
Master One Health	Europe	UK	University of Edinburgh	Royal (Dick) School of Veterinary Sciences	online	3-6 years	graduate	2	ES, EID
MSc in Global Health: Pathogens and Policy	Europe	UK	Royal Holloway university of london	School of Biological Science	in-person	1-2 years	graduate	2	CC, EID
MSc One Health: ecosystems, humans and animals	Europe	UK	Royal Veterinary College (RVC) /London School of Hygiene & Tropical Medicine (LSHTM)	Vet Med	in-person or online	1-2 years	graduate	1	EID

Supplementary Material: Institutions with education in biodiversity and health interlinkages									
*ES - Ecosystem services, CC - Climate change adaptation and disaster risk reduction, EID - emerging infectious disease, WB - physical, mental and cultural well-being, Pharma - Traditional medicine and biodiversity for pharmaceuticals, MB - microbial diversity and non-communicable diseases									
**Program/Course Title (language of instruction if not English)**	**Region**	**Country**	**Institution**	**Faculty**	**Delivery**	**Length**	**Target group**	**#of Concepts**	**Concepts Covered**
MSc Public Health (Environment and Health)	Europe	UK	RVC/LSHTM	vet med	in person or online	1-2 years	graduate	2	ES, CC
MPH Concentrations: Global Environmental Sustainability and Health; Food, Nutrition and health	North America	USA	Johns Hopkins	Bloomberg School of public health	in-person online	1 year 2 years	graduate	2	ES, CC
undergraduate courses in environment and human health, planetary health; host of planetary health alliance	North America	USA	Harvard University	Centre for the Environment	in-person	1 semester	undergraduate	2	ES, CC, WB
Undergraduate One Health minor	North America	USA	Penn State University	College of Agricultural Sciences	in-person	4 years (as part of undergraduate degree)	undergraduate	1	ES
Undergraduate Bachelor of One Health and One Health minor	North America	USA	Fontbonne University	College of Arts and Sciences	in-person	4 years	Undergraduate	3	EID, CC, ES
Master in Public Health Concentration in Food Security and Nutrition	North America	USA	George Mason University	College of Health and Human Services	in-person	2 years	graduate	1	ES
Hosts yearly One Health Conference in Collaboration with local Public Health office	North America	USA	Baylor University	College of Medicine	in-person	3 days	professionals	3	WB, EID, CC
Master of Public Health	North America	USA	University of Vermont	College of Medicine	online	1-3 years	graduate	2	EID, CC
MPH One Health	North America	USA	University of Arizona	College of Public Health	in-person	2 years	graduate	2	ES, CC
One Health courses throughout Veterinary degree program	North America	USA	Lincoln Memorial University (LMU)	College of Veterinary Medicine	in-person	4 years	Vet	3	WB, CC, ES
various one health undergraduate courses	North America	USA	Texas A&M University	College of Veterinary Medicine	in-person	1 semester	undergraduate	2	CC, EID
summer study abroad Global One Health program	North America	USA	Texas A&M University	College of Veterinary Medicine	in-person	10 weeks	vet	2	CC, EID
Global One Health Initiative (with Tufts University) MPH Global Environmental Health	North America	USA	University of Minnesota	College of Veterinary Medicine	in-person	2 years	graduate	3	CC, EID, ES
Master of Science in Conservation Medicine, One Health Workforce program (SEAOHUN and AFROHUN)	North America	USA	Tufts University	Cummings School of Veterinary Medicine; Clinical and Translational Science Institute	in-person	1 year	graduate, vet	2	EID, ES, CC
Ungraduate Major in Global Health and Environment	North America	USA	Washington University in St. Louis	Department of Anthropology	in-person	4 years	undergraduate	1	ES
Master of Science in Climate and Health (new for 2020)	North America	USA	University of Miami	department of public health science	in-person	2 years	graduate	1	CC
Human Health and the Environment course	North America	USA	Lehigh University	Faculty of Earth and Environmental Sciences	in-person	1 semester	undergraduate	3	CC, ES, MB
One Health clinical elective	North America	USA	University of Washington	Faculty of Medicine	in-person	1 semester	medicine	2	EID, WB
Population, Health and the Environment online course (also available in French and Spanish)	North America	USA	Population Reference Bureau/USAID	Global Health e-Learning Center	online	own pace	anyone	1	CC
PhD in Environmental and Planetary Health	North America	USA	City University of New York	Graduate School of Public Health and Health Policy	in-person	5-6 years	post-graduate	2	ES, MB
Master of Science in Global Health	North America	USA	University of California, San Francisco	Institute for global health science	in-person	1 year	graduate	2	CC, ES

Supplementary Material: Institutions with education in biodiversity and health interlinkages									
*ES - Ecosystem services, CC - Climate change adaptation and disaster risk reduction, EID - emerging infectious disease, WB - physical, mental and cultural well-being, Pharma - Traditional medicine and biodiversity for pharmaceuticals, MB - microbial diversity and non-communicable diseases									
**Program/Course Title (language of instruction if not English)**	**Region**	**Country**	**Institution**	**Faculty**	**Delivery**	**Length**	**Target group**	**#of Concepts**	**Concepts Covered**
Climate Change and Health online course	North America	USA	Next Gen U	n/a	online	own pace	anyone	1	CC
MPH in Environmental Health	North America	USA	Emory University	Rollins School of Public Health	in-person	2years	graduate	2	EID, ES
Online One Health graduate certificate	North America	USA	Auburn University	School of Forestry and Wildlife Sciences	online	own pace	Graduate	2	EID, ES
Minor in Global Environmental Sustainability	North America	USA	Colorado State University	School of Global Environmental Sustainability One Health Institute	in-person	4 years (as part of undergraduate degree)	undergraduate	2	CC, ES
One Health Summer Program (new in 2020)	North America	USA	Colorado State University	School of Global Environmental Sustainability One Health Institute	online	8 weeks	vet	1	CC
MPH Concentration: Environmental Public Heath Sciences	North America	USA	New York University	School of Global Public Health	in-person	2 years	graduate	1	CC
Master of Public Health	North America	USA	University of Missouri	School of Health Professions	in-person or online	2 years	graduate	2	ES, CC
Undergraduate major: Ecosystems and Human Impact	North America	USA	Stony Brook University	School of Marine and Atmospheric Sciences	in-person	4 years	undergraduate	3	ES, WB, CC
Undergraduate One Health Minor, One Health Center	North America	USA	Berry College	School of Mathematical and Natural Sciences	in-person	4 years	undergraduate	3	EID, WB, CC
Global Leaders and Innovators in Human and Planetary Health course (medicine elective)	North America	USA	Stanford University	School of Medicine	in-person	1 semester	medicine	1	ES
UNC One Health elective course	North America	USA	University of North Carolina Chapel Hill	School of Medicine	in-person	1 semester	medicine	2	EID, CC
Global Health Certificate Program	North America	USA	Princeton University	School of Public and International Affairs	in-person	2 years (as part of 4 year undergrad)	undergraduate	1	ES
MPH Environmental Health Sciences	North America	USA	Columbia University	School of Public Health	in-person	2 years	graduate	2	ES, CC
MPH Global Health and Environment	North America	USA	University of California, Berkeley	School of Public Health	in-person	2 years	graduate	1	CC
Graduate Certificate in Climate Change and Health	North America	USA	University of Washington	School of Public Health	in-person	2 semesters	graduate	1	CC
MPH One Health	North America	USA	University of Washington	School of Public Health	in-person	2 years	graduate	3	EID, ES, WB
MPH in Environmental Health	North America	USA	University of Washington	School of Public Health	in-person	2 years	graduate	1	ES
Graduate certificate in One Health	North America	USA	University of Washington	School of Public Health	in-person	2 semesters	graduate	3	EID, ES, WB
undergraduate Global Population, Health, and Environment course	North America	USA	University of California, Davis	School of Veterinary Medicine	online	1 semester	undergraduate	3	EID, ES, CC
Master of Preventive Veterinary Medicine	North America	USA	University of California, Davis	School of Veterinary Medicine	in-person	1 year	Vet	2	EID, ES
MPH concentration courses	North America	USA	Harvard University	TH Chan School of Public Health	in-person	2 years (program)	graduate	2	Pharma, WB

Supplementary Material: Institutions with education in biodiversity and health interlinkages									
*ES - Ecosystem services, CC - Climate change adaptation and disaster risk reduction, EID - emerging infectious disease, WB - physical, mental and cultural well-being, Pharma - Traditional medicine and biodiversity for pharmaceuticals, MB - microbial diversity and non-communicable diseases									
**Program/Course Title (language of instruction if not English)**	**Region**	**Country**	**Institution**	**Faculty**	**Delivery**	**Length**	**Target group**	**#of Concepts**	**Concepts Covered**
Postdoctoral Fellowships in Planetary Health	North America/Europe	USA/UK	Stanford University/LSHTM	School of Medicine	in-person	2 years	post-doc	2	ES, CC
One Health Modules	Africa	various countries	AFROHUN	N/A	online	own pace	professionals	4	EID, WB, ES, CC
